# *Mycobacterium tuberculosis* complex lineages and drug resistance patterns among tuberculosis patients with or without diabetes mellitus in southern Ghana

**DOI:** 10.1371/journal.pone.0338498

**Published:** 2025-12-15

**Authors:** Emelia Konadu Danso, Prince Asare, Amanda Yaa Tetteh, Phillip Tetteh, Augustine Asare Boadu, Ivy Naa Koshie Lamptey, Augustina Angelina Sylverken, Kwasi Obiri-Danso, Jane Sandra Afriyie-Mensah, Abraham Adjei, Dorothy Yeboah-Manu

**Affiliations:** 1 Department of Bacteriology, Noguchi Memorial Institute for Medical Research, University of Ghana, Accra, Ghana; 2 Department of Theoretical and Applied Biology, Kwame Nkrumah University of Science and Technology, Kumasi, Ghana; 3 University of Ghana Medical School, University of Ghana, Accra, Ghana; 4 Department of Chest Diseases, Korle-Bu Teaching Hospital, Accra, Ghana; St Petersburg Pasteur Institute, RUSSIAN FEDERATION

## Abstract

Drug-resistant (DR) tuberculosis (TB) and diabetes mellitus (DM) are intersecting epidemics that complicate management of both diseases and worsen patient outcomes. We conducted a prospective cohort study of 758 GeneXpert-confirmed pulmonary TB patients, of whom 75 had DM. Demographic, clinical, radiographic, and anthropometric data were collected at baseline. Sputum samples were cultured for mycobacterial isolation, and the obtained isolates were characterized for *Mycobacterium tuberculosis* complex (MTBC) lineage and drug-susceptibility testing using spoligotyping and microplate alamar blue assay. The TB-diabetes (TB-DM) comorbid cohort was older [TB-DM: 53/75 (70.7%) vs. 241/683 (35.3%) aged 41–60 years) (p < 0.001), included a higher proportion of females [TB-DM: 31/75 (41.3%) vs. TB-only: 150/683 (22.0%), p < 0.001], and had greater mean BMI (TB-DM: 23.36 ± 0.99 vs. TB-only: 19.97 ± 0.45 kg/m², p = 0.003). Analysis of 501 (TB-only: 448, TB-DM: 53) MTBC isolates revealed that TB-DM patients are more likely to get TB caused by L6 [TB-DM: 10/53 (18.9%) vs. TB-only: 37/448 (8.3%), p = 0.022] compared to the general TB population Lineage 4 [TB-DM: 36/53 (67.9%) vs. TB-only: 362/448 (80.8%), p = 0.046], Mycobacterial strains from TB-DM exhibited higher isoniazid mono-resistance [TB-DM: 15/50 (30.0%) vs. 42/288 (14.6%), p = 0.012] and harbored more multidrug-resistant TB [TB-DM: 5/50 (10.0%) vs. TB-only: 16/288 (5.6%), p = 0.215] although this did not reach statistical significance. These findings indicate that DM not only predisposes individuals to TB but may also shift the spectrum of infecting lineages and promotes the emergence of DR strains. Integrated TB-DM screening, lineage-aware diagnostics, and tailored treatment protocols are urgently needed in high-burden settings to address this dual threat.

## Introduction

Drug-resistant (DR) mycobacterial strains present a significant challenge to the global tuberculosis (TB) control efforts. In 2023, the World Health Organization (WHO) reported an estimated 400,000 cases of multidrug-resistant TB (MDR-TB), highlighting the ongoing threat posed by resistant strains [1]. Concurrently, the global burden of diabetes mellitus (DM) is increasing, particularly in regions with a high TB prevalence [2]. Epidemiological studies suggest that DM is not only a risk factor for TB development but also DR-TB [3,4].

DM has been associated with increased TB severity due to impaired immune responses, including dysfunctional macrophage activity, altered cytokine production, and chronic hyperglycaemia, all of which contribute to delayed bacterial clearance [5–7]. Moreover, TB patients with DM have been found to exhibit higher rates of treatment failure, relapse, and mortality compared to non-diabetic TB patients [8]. Recent meta-analyses indicate that DM nearly doubles the risk of MDR-TB, suggesting a possible link between hyperglycaemia and the selection of DR *Mycobacterium tuberculosis* complex (MTBC) strains [9,10].

Tuberculosis is treatable; the WHO-endorsed first-line treatment protocol is the Directly Observed Treatment Short Course (DOTS) which spans six months and is divided into two phases. Drug-resistant TB can be caused by inadequate treatment and poor adherence, leading to genetic mutations in the bacteria [11]. There are many forms of DR-TB: – MDR-TB denotes a strain of MTBC that is resistant to at least INH and RIF. Extensively DR-TB (XDR-TB) refers to TB disease caused by an MDR/RIF resistant-TB strain that is additionally resistant to any fluoroquinolone and a group A drug that include bedaquiline and linezolid [11]. These resistant strains reduce the effectiveness of treatment, potentially threatening TB to become an untreatable disease [12].

Tuberculosis in mammals is mainly caused by the MTBC, which includes human-adapted (hMTBC), and animal adapted (aMTBC) species. The hMTBC, consists of *Mycobacterium tuberculosis sensu stricto* (Mtbss, lineages 1, 2, 3, 4, 7 and 8) and *M. africanum* (Maf, lineages 5, 6 and 9). Moreover, the aMTBC including *M. bovis* can also cross infect humans [13]. The distribution of MTBC lineages also varies among patient populations, with some studies suggesting that specific lineages may be less virulent and more prevalent among individuals with immune downregulation [14,15].

This study sought to examine the relationship between DM, DR-TB, and MTBC lineage distribution by analysing clinical isolates from TB patients with and without DM. By characterizing drug susceptibility profiles in these isolates, we aimed to provide insights into whether DM is associated with a predisposition to resistant TB strains or specific MTBC lineages, which could have significant implications for TB control strategies in high-burden settings.

## Method

### Study design and participant recruitment

This was a cross-sectional study, in which GeneXpert confirmed pulmonary TB patients were recruited from four public health facilities (in Accra, Ghana) before initiation of anti-TB therapy from 20th October 2020–30th November 2023.

Ethical approvals for all study protocols were obtained from the Noguchi Memorial Institute for Medical Research (NMIMR) Institutional Review Board in Ghana (FWA00001824/2019). Informed consent (written) was sought from each participant before enrolment into the study. The study’s purpose, objectives, potential risks, benefits, and confidentiality measures were explained to all participants. Data collection was facilitated through a standardized questionnaire designed to gather both demographic and clinical information. This questionnaire included details such as age, gender, marital status, employment status, and a comprehensive description of the participant’s residence. Additionally, anthropometric measurements, glucose levels, and any existing clinical diagnoses were recorded. Moreover, vital signs, including weight, body mass index (BMI), blood sugar levels, and blood pressure were measured. In accordance with the American Diabetes Association (ADA) criteria, a finger-prick blood (using ACCU-CHEK Guide Test Strips) was used to assess the random blood glucose (RBG) levels of all recruited study participants using a glucometer (ACCU-CHEK, Roche Diabetes Care Limited, Burgess Hill, UK). Patients with RBG ≥ 7 mmol/L had their blood drawn for a confirmatory test, glycated haemoglobin (HbA1c). Patients with HbA1c≥6.5% were classified as diabetic (TB-DM) cohort and those with HbA1c<6.5% were TB-only cohort. Chest radiographs were analysed by a pulmonologist, who identified lesions as alveolar infiltrates and cavities. In line with routine practice recommended [16], all TB patients recruited for the study were screened for HIV infection.

### Inclusion and exclusion criteria

TB only (TB patients without diabetes) cohort refers to newly diagnosed TB cases above 18 years with no known immune suppression illness, or not on any immunosuppressive drugs that predispose one to TB, who consented to the study and had not yet been initiated on anti-TB medications. TB-DM (TB patients with diabetes) cohort was made up of participants with co-morbidity of TB and DM who consented to the study and had not yet initiated anti-TB medication. In line with Ghana’s National Tuberculosis Control Programme recommendations, clinicians initiated anti‑TB therapy within 24 hours of diagnosis for every participant [17].

Children below the ages of 18 years, HIV/AIDS patients, individuals on immunosuppressive drugs, pregnant women with TB, or TB-DM were excluded from the study.

### Sample collection and transport

A sputum sample collected early in the morning from TB patients visiting the selected health facilities, before chemotherapy, was immediately placed in labelled 100 mL wide-mouth containers. Each sample was securely capped, sealed with parafilm, and packed in a cold box with ice and transported according to the WHO guidance on regulations for the transport of infectious substances [18] to the Bacteriology Department of the NMIMR laboratories in Accra for analysis.

### Laboratory investigations

Microbiological investigations were performed using baseline sputum samples for microscopy and culture. All analyses were performed aseptically in a biosafety level three physical containment laboratory of the NMIMR-University of Ghana, Accra-Ghana.

### GeneXpert identification

The GeneXpert MTB/RIF assay was used to detect *Mycobacterium tuberculosis* and rifampicin resistance in sputum samples at the point of care. Sputum was mixed with the GeneXpert reagent (2:1), shaken for 15 minutes to liquefy and deactivate bacteria, then loaded into a sealed cartridge. The GeneXpert machine automatically performed DNA extraction, amplification, and real-time PCR detection, displaying results for TB presence and rifampicin resistance.

#### Culture.

Sputum samples were decontaminated with equivalent volumes of 5% (w/v) oxalic acid and later inoculated on two pairs of Lowenstein-Jensen (LJ) media slants supplemented with glycerol (1 pair of LJ media slants) and pyruvate (1 pair of LJ media slants) in separate glass tubes. The inoculated tubes were incubated at 37 °C and observed weekly for confluent mycobacteria growth for 3 months [19].

#### Smear microscopy.

Sputum smears were prepared directly from the decontaminated resuspended pellet on clean well-labelled microscope slides and stained using the Ziehl-Neelsen staining method. The stained slides were then examined under the microscope and quantified using a grading system earlier described [20].

### Identification of *Mycobacterium tuberculosis* complex lineages

Mycobacteria isolates were harvested, and DNA was extracted by boiling isolates at 95 °C for 1 hour to release mycobacterial nucleic acid material into the supernatant. The supernatant of the heat killed isolates was used for further molecular analysis. The obtained nucleic acid of the isolates was initially confirmed as a member of the MTBC by amplification of the insertion sequence 6110 (IS*6110*) using polymerase chain reaction (PCR) as previously described [21]. The confirmed isolates were then genotyped using spacer oligonucleotide typing (spoligotyping) by amplification of the direct repeat region and subsequent hybridization onto a film [22]. The obtained binary data indicates the presence or absence of spacers in the DR region. The lineages, sub-lineages and their shared international type (SIT) numbers were assigned using identification by similarity search option of the MIRU-VNTR*plus*, SITVIT2 and *Mycobacterium bovis* spoligotype web databases to determine the infecting lineages and sub-lineages [23]. The phylogenetic tree was constructed using the MIRU-VNTR*plus* database and visualized using ITOL v.6 [24].

### Drug susceptibility testing

The microplate alamar blue assay (MABA) was performed to determine minimum inhibitory concentrations of isoniazid (INH) and rifampicin (RIF) as described previously (Franzblau et al., 1998; Otchere et al., 2016), and/or the GenoType MTBDR*plus* line-probe assay (Hain Lifescience, Germany) following the manufacturer’s protocol (Barnard et al., 2008). In the MABA, serial two-fold dilutions of each drug were prepared in Middlebrook 7H9-glycerol-casitone (7H9GC). The 7H9GC broth was supplemented with OADC and filter sterilized 10% tween 80 within 96-well plates, inoculated with MTBC isolates or H37Rv (reference strain) standardized to McFarland No. 1, and incubated at 37 °C for seven days. After incubation, an equal volume of alamar blue reagent with 0.05% Tween 80 was added and incubated for another 24 hours. Here, wells that remained blue indicated growth inhibition, while a blue-to-pink colour change denoted the presence of viable bacteria. The lowest concentration preventing colour change was recorded as the MIC.

### Statistical analysis

During recruitment and treatment, each participant’s information was entered into Microsoft Access (Office 365) and then cross-checked against the original structured questionnaires to identify and remove any inconsistencies or duplicate entries. Analyses were performed using GraphPad Prism version 9.4.1 and Stata version 14.2. Statistical comparisons employed Fisher’s exact test and the chi-square test as appropriate, with p < 0.05 considered the threshold for significance.

## Results

**Fig 1:**
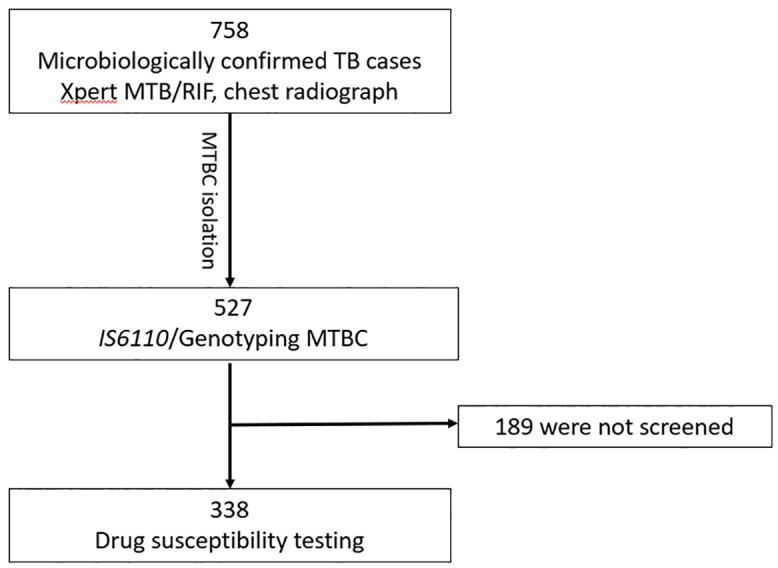
Flowchart of included and excluded participants and workflow. A total of 758 (including 75 TB-diabetes participants) participants with bacteriologically confirmed pulmonary tuberculosis were enrolled in the study. Among these, 527 isolates were successfully genotyped and characterized. Of the genotyped isolates, 338 were subsequently subjected to phenotypic drug susceptibility testing for further analysis.

Table 1 summarizes the characteristics of the study participants. This investigation was part of a larger study that enrolled 758 GeneXpert-confirmed newly diagnosed pulmonary TB patients, of whom 75 (9.9%) had TB with diabetes mellitus. Of the 758 samples processed, we successfully obtained MTBC isolates and genotyped 527 which were further characterised. Of the 527 genotyped isolates, 338 were subsequently subjected to drug susceptibility testing (Fig 1). Out of the 758 participants, the majority were males 577/758 (76%), with a significantly higher proportion in the TB-only group 533/683 (78%) compared to the TB-DM group 44/75 (58.7%, p < 0.001). Female participants constituted 181/758 (24%) overall, with a notably higher proportion in the TB-DM group 31/75 (41.3%) than in the TB-only group 150/683 (22%). In terms of age distribution, there was a statistically significant difference between the groups (p < 0.001). The majority of TB-DM patients were aged 41–60 years 53/75 (70.7%), while TB-only cases were more spread across the younger age groups, particularly 26–40 years 251/683 (36.7%) and 18–25 years 121/683 (17.8%). There were no TB-DM participants in the 18–25 age group. Chest X-ray data (available for 235 participants) showed that most individuals in both groups had abnormal findings. All TB-DM patients had abnormal chest radiographs compared to 97.4% in the TB-only group (p = 0.059). Body weight and BMI were significantly higher in the TB-DM group (62.61 ± 2.48 kg and 23.36 ± 0.99 kg/m², respectively) compared to the TB-only group (55.14 ± 1.16 kg and 19.97 ± 0.45 kg/m²), with p-values of 0.008 and 0.003, respectively.

**Table 1:** Participants demographic and clinical data.

Variables	N (%)	TB-only, n (%)	TB-DM, n (%)	p-value
**Gender**	758			
**Male**	577 (76)	533 (78)	44 (58.7)	<0.001
**Female**	181 (24)	150 (22)	31 (41.3)	
**Age**	758			
**18-25**	121 (16.0)	121 (17.8)	0 (0.0)	<0.001
**26-40**	261 (34.4)	251 (36.7)	10 (13.3)	
**41-60**	294 (38.8)	241 (35.3)	53 (70.7)	
**Over 60**	82 (10.8)	70 (10.2)	12 (16.0)	
**Chest X-ray**	235			
**Normal**	5 (2.1)	5 (2.6)	0 (0.0)	0.059
**Abnormal**	230 (97.9)	189 (97.4)	41 (100)	
**Body weight (Kg)**	758	55.14 ± 1.16	62.61 ± 2.48	0.008
**BMI (Kg/m**^**2**^)	758	19.97 ± 0.45	23.36 ± 0.99	0.003
**Smoked**	758			
Yes	259 (34.2)	246 (36)	13 (17.3)	0.001
**Alcohol**	757			
Yes	432 (57)	390 (57.1)	42 (56)	0.790
**Drug abuse**	757			
Yes	149 (19.7)	143 (20.9)	6 (8)	0.001

This table summarizes the demographic and clinical data of study participants stratified by cohort (TB-only and TB-DM). Data are presented as counts and percentages [n (%)] for categorical variables and as mean ± standard error of mean (SEM) for continuous variables. P-values were calculated using Fisher’s exact test for categorical variables and Student’s t-test for continuous variables. Significant associations (p < 0.05) are shown in bold.

Regarding lifestyle factors, more than half of the participants consumed alcohol, one-third smoked and less than a quarter abused drugs (Table 1).

### Genotype distribution among cohorts

Among the 527 MTBC isolates successfully identified to species level, *M. tuberculosis* sensu stricto (MTBss) was the predominant species, accounting for 439/527 (83.3%) of all cases. This species was more common among TB-only patients 378/471 (80.3%) compared to those with TB-DM [38/56 (67.9%), p = 0.037]. *M. africanum* was found in 85/527 (16.1%) of all cases, with a higher proportion in TB-DM patients 15/56 (26.7%) than TB-only patients 70/471 (14.9%, p = 0.022). *M. bovis* was rare, identified in only three TB-only patients 3/471 (0.6%) and absent in the TB-DM group.

Of the 527, 23 MTBC isolates were not assign to a lineage by spoligotyping. Phylogenetic analysis using MIRU-VNTR*plus* showed that the majority (19/23) clustered with Lineage 4 sub-lineages, specifically UgandaI (n = 11), Haarlem (n = 5), Ilama (n = 2), and Cameroon (n = 1). The remaining isolates included two assigned to Lineage 3 (Delhi/CAS), one to Lineage 1 (EAI), and one identified as *M. caprae* (not shown). All except three of these 23 isolates were from the TB-only cohort; the three in the TB-DM group included two Uganda I and one *M. caprae.* After excluding the three *M. bovis* strains and the 23 isolates with undetermined lineages, a total of 501 (TB-only: 448, TB-DM: 53) strains were included in the subsequent analysis.

**Fig 2:**
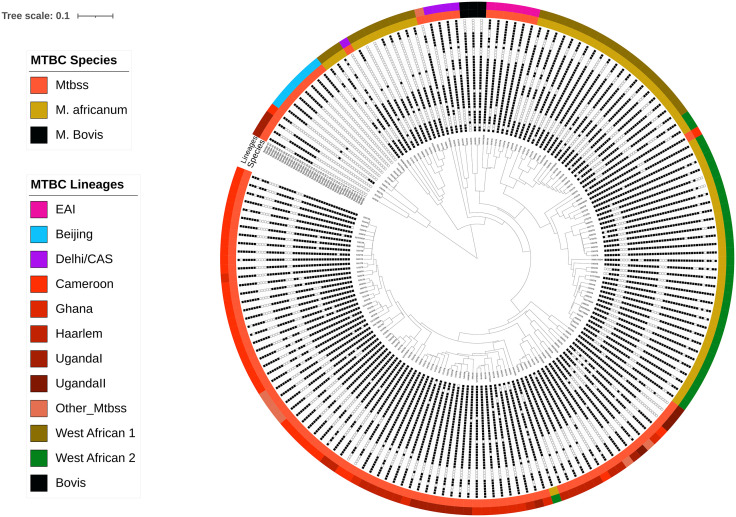
Phylogenetic tree based on spoligotyping of *Mycobacterium tuberculosis* complex isolates. It illustrates the *Mycobacterium tuberculosis* complex species, their corresponding lineages and spoligotype patterns. Of the 501 spoligotypes, 190 belonging to the Cameroon sub-lineage and 50 to the Ghana sub-lineage with identical patterns collapsed to enhance visual clarity of the phylogenetic tree.

**Fig 3:**
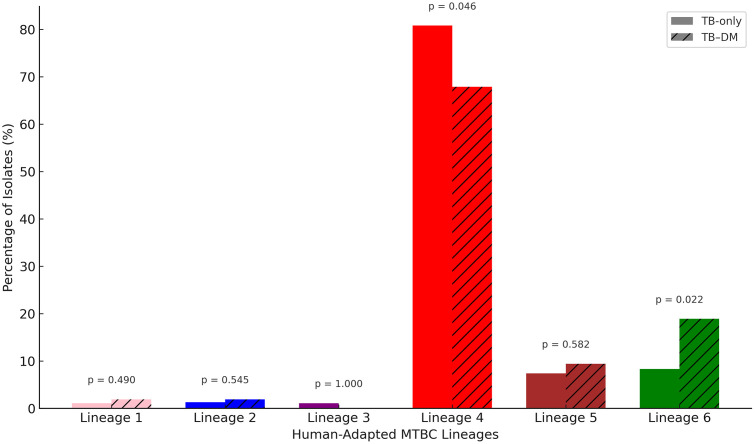
Distribution of MTBC lineages by cohort. Clustered bar chart comparing MTBC lineage distributions between TB-only and TB-DM cohorts, with p-values indicated above each lineage pair. The colours depict the unique internationally recognized lineage colour scheme.

Human-adapted lineage typing was successfully done for 501 isolates. Lineage 4 was the most prevalent, representing 398/501 (79.4%) of isolates overall (Figs 2 and 3). It was more frequent among TB-only patients 362/448 (80.8%) than in those with TB-DM 36/53 (67.9%, p = 0.046). Lineage 6 was found in 9.4% of isolates, with a higher proportion among TB-DM cases 10/53 (18.9%) compared to TB-only cases 37/448 (8.3%, p = 0.022) (Figs 2 and 3). Other lineages (Lineages 1, 2, 3, and 5) occurred at lower frequencies and showed no significant differences between the two groups (Fig 3).

**Fig 4:**
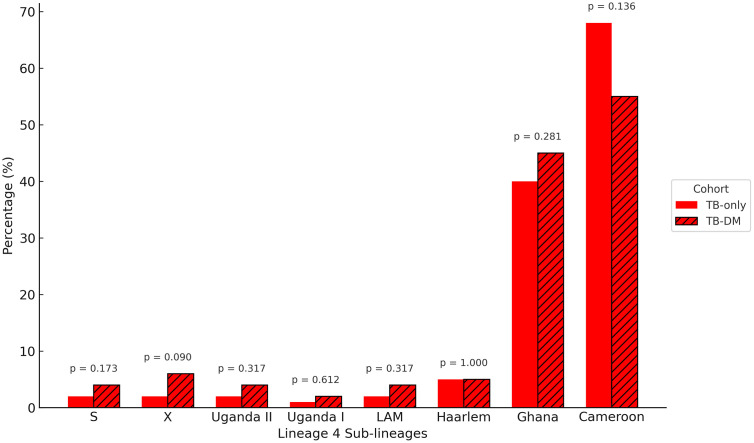
Distribution of lineage 4 sub-lineages by cohorts. Clustered bar chart comparing TB-only and TB-DM cohorts across lineage 4 sub-lineages, with p-values displayed above each sub-lineage pair.

Within lineage 4 (n = 398), the most common sub-lineage was the Cameroon genotype, accounting for 268/398 (67.3%) of cases. This was more prevalent among TB-only patients 248/362 (69%) compared to TB-DM patients 20/36 (55.5%, p = 0.136) although this was not statistically significant (Figs 2 and 4). The Ghana sub-lineage followed at 82/398 (20.6%), with a slightly higher proportion among TB-DM patients 10/36 (27.7%) than TB-only 72/362 (20%, p = 0.281), but this did not reach statistical significance. Other sub-lineages such as Haarlem, LAM, Uganda I and II, X, and S were less common (Fig 4).

Phylogenetic analysis of 501 MTBC isolates, revealed clear lineage and sub-lineage clustering. Within Lineage 4, distinct clades were formed by the Cameroon and Ghana families, both of which showed tight and coherent clustering indicative of local clonal expansion (Fig 2).

The *M. africanum* (L5 and L6) isolates were phylogenetically distinct, appearing on separate branches rather than forming a single cluster. This indicates the circulation of multiple Maf strains within the population. Both TB-DM and TB-only isolates were distributed across major lineages. However, TB-DM isolates were more frequently found within Lineage 6 and the Ghana sub-lineage, while TB-only isolates predominantly clustered within Lineage 4 (Cameroon genotype). These patterns suggest possible lineage-specific differences between TB-DM and TB-only patients (Fig 2).

### Drug susceptibility profile among the participants

A total of 338 MTBC isolates were tested for drug susceptibility, comprising 288 isolates from TB-only patients and 50 from individuals with TB-DM comorbidity. Among TB-only patients, 225 (78.1%) isolates were fully susceptible to both isoniazid and rifampicin, compared to only 28 (56%) among TB-DM patients, indicating a significantly lower rate of drug-susceptible TB in the TB-DM group (p < 0.002) (Table 2).

**Table 2:** Drug susceptibility patterns among TB-only versus TB-DM participants.

Type of TB drug resistant	TB only, n (%)	TB-DM, n (%)	p-value
Sensitive	225 (78.1)	28 (56)	0.002
Isoniazid mono resistant	42 (14.6)	15 (30)	0.007
Rifampicin mono resistant	5 (1.7)	2 (4.0)	0.299
Multidrug resistant-TB	16 (5.6)	5 (10)	0.215
Total	288 (85.2)	50 (14.8)	

This table presents the distribution of TB drug resistance profiles among participants stratified by diabetes status. The data highlight differences in the proportions of resistant isolates between TB-only and TB-DM cohorts, suggesting significantly higher burden of isoniazid mono-resistance. N = 338 [TB only = 288 (85.2%): TB-DM = 50 (14.8%)]

Isoniazid mono-resistance was observed in 42/288 (14.6%) of isolates from TB-only patients, whereas the proportion was markedly higher among TB-DM patients at 15/50 (30%), (p = 0.012). Rifampicin mono-resistance was relatively low in both groups, detected in 5/288 (1.7%) of TB-only cases and 2/50 (4%) of TB-DM cases, with no significant difference observed (p = 0.277) (Table 2).

Multidrug-resistant TB (MDR-TB), defined as resistance to both isoniazid and rifampicin, was identified in 16 of 288 (5.6%) TB-only patients and in 5 of 50 (10%) TB-DM patients (p = 0.215) (Table 2), suggesting a higher burden of drug resistance in the TB-DM population, although the difference did not reach statistical significance.

## Discussion

Tuberculosis and DM are two intersecting global health threats, with indication that each could influence the clinical course, treatment outcomes, and epidemiology of the other. As the dual burden of TB-DM comorbidity increases, especially in high TB prevalence settings, it becomes critical to understand whether DM affects TB disease presentation, bacterial strain distribution, and DR patterns. This study therefore examined the relationship between DM, clinical epidemiology of patients, DR and infecting MTBC lineages by analyzing clinical isolates from TB patients with and without DM.

Children under 18 years were excluded because the immune response, pharmacokinetics of TB drugs, and disease progression differ significantly from adults, which could introduce variability not related to diabetes [25]. People living with HIV/AIDS were excluded because HIV-associated immunosuppression independently affects TB progression, drug metabolism, and resistance patterns, making it difficult to isolate the specific impact of diabetes [26]. Similarly, individuals receiving immunosuppressive therapy were excluded because such treatments alter immune function and may confound the relationship between diabetes and TB drug resistance [27]. Pregnant women were excluded for both ethical and biological reasons: pregnancy alters immune and metabolic functions, and certain TB drugs are contraindicated or modified during pregnancy, which could affect treatment response and outcomes [25].

These exclusions were therefore necessary to ensure a more homogeneous study population and to accurately assess the independent effect of diabetes on drug-resistant TB. The significantly lower proportion of males and younger individuals in the TB-DM group is consistent with existing literature suggesting that TB-DM patients tend to be older and more evenly distributed across genders compared to TB-only patients, likely due to the age-related risk of DM development [3]. The striking age disparity and in particular the absence of TB-DM comorbidity among individuals under 25 years, reinforces the well-established association between increasing age and the risk of type 2 DM [28]. The observed higher BMI and body weight among TB-DM patients are also in line with known metabolic characteristics of DM. While TB typically leads to weight loss, the elevated baseline body weight in the TB-DM group likely reflects the underlying metabolic condition such as insulin resistance, increased adiposity and impaired glucose metabolism. Nonetheless, it’s worth noting that higher BMI in TB-DM does not necessarily confer protection for TB, as DM can impair immune responses, potentially worsening TB outcomes [5]. These observations highlight the need for targeted screening for DM in TB patients over 40, and in obese TB patients particularly in high TB settings.

Abnormal chest radiographs were found in nearly all participants, representative for active pulmonary TB. However, the slightly higher frequency of abnormal radiographs in TB-DM patients may point to more extensive or severe disease, consistent with previous reports suggesting delayed immune response and atypical radiologic patterns in TB-DM patients [5,7]. Significantly lower rates of smoking and drug abuse in TB-DM participants may reflect age-related behavior trends or increased healthcare engagement due to DM. Conversely, the higher rates of these risk behaviors in the TB-only group points to social determinants of health and lifestyle factors as important contributors to TB burden. These findings underscore the importance of integrating diabetes screening into TB programs, particularly for older patients. They also highlight the need to tailor public health strategies to address comorbidity profiles and lifestyle risks specific to TB and TB-DM populations.

We found differences in prevalence of MTBC genotypes between TB-only and TB-DM patients, underscoring a possible influence of host metabolic status on strain prevalence and adaptation. The higher proportion of *M. tuberculosis* sensu stricto among TB-only patients aligns with its global predominance as the primary causative agent of TB [29]. The lower frequency in TB-DM patients, alongside a higher proportion of *M. africanum* suggest a potential niche preference or host-pathogen interaction dynamic in diabetic individuals. *M. africanum* is typically endemic in West Africa and has been reported to be less virulent than *M. tuberculosis* [14], but its increased frequency in TB-DM patients could point toward host metabolic compromise favoring infection by less aggressive strains. Furthermore, the significantly higher proportion of Lineage 6, raises interesting questions about whether more West-Africans harbor the less virulent L6 as latent infection. Lineage 6 has been associated with immune modulation and slower disease progression [15], which may make it more likely to persist or reactivate in metabolically compromised hosts like those with HIV infection and DM.

Our findings indicate that TB-DM isolates have significantly higher resistance to anti-TB drugs compared to TB-only isolates. The chronic hyperglycemic state in diabetic patients may impair both innate and adaptive immunity, thereby reducing the efficacy of treatment and facilitating the emergence of MDR-TB strains. Furthermore, differences in host metabolism between the groups might lead to lower effective drug concentrations in TB-DM patients, accelerating the selection of resistant clones [10]. Moreover, some reports suggest that impaired immune response in DM may alter drug pharmacokinetics, potentially leading to suboptimal bacterial clearance [7] and selection of resistant mutants [3,8]. The higher rate of resistance among TB-DM cases underlines the need for early and robust diagnostic measures in patients with DM.

The significant difference in INH mono-resistance between TB-only and TB-DM isolates is particularly concerning. Isoniazid is one of the cornerstone drugs in TB treatment, and resistance to it can be an early indicator of MDR. This observation aligns with findings from Tegegne et al. (2018), which reported that DM increases the odds of developing INH resistance. Impaired drug absorption or altered metabolism in diabetic patients may contribute to this phenomenon, leading to sustained low-level exposure that promotes the emergence of resistance [10]. It is therefore not surprising that we found a higher prevalence of MDR-TB in the TB-DM group compared to TB-only isolates, although this difference did not reach statistical significance.

Although RIF mono-resistance was observed in both cohorts, the difference was not statistically significant. This could be due to the overall low prevalence of RIF resistance at baseline, which is consistent with other studies indicating that RIF resistance often occurs in combination with INH resistance rather than as a mono-resistant pattern [11]. Therefore, while RIF resistance is critical for the diagnosis of MDR-TB, its baseline occurrence as a standalone resistance may not be as strongly influenced by DM.

To better understand the impact of chronic hyperglycemia in TB-DM patients on drug exposure, an ongoing component of the current study is investigating pharmacokinetic/pharmacodynamic parameters and the impact of glycemic control on TB treatment response.

A limitation of this study is the absence of data on the average duration of diabetes mellitus prior to TB diagnosis in the TB-DM group. Our strength is that this study benefits from a well‑characterized cohort and is one of the few to offer comprehensive data on strain diversity, drug susceptibility, and host metabolic status in a West African setting.

In conclusion, this study highlights important demographic, genotypic, and resistance-related differences between TB-only and TB-DM patients. Diabetes was associated with infection by distinct MTBC lineage and significantly higher rate of drug resistance, particularly isoniazid. These findings emphasize the urgent need for integrated TB-DM programs that include routine diabetes screening, tailored diagnostic approaches, and a more aggressive management.
